# Assassination and "Militantism."

**Published:** 1913-03-29

**Authors:** 


### ASSASSINATION AND "MILITANTISM."

The murder of the King of Greece is an extraordinary example of the utter absence of any of those compelling motives which at times may seem to justify the resort to extreme measures on the part of oppressed subjects. It was the crime of a madman. It is well to urge the point, for there is a connection, more vital than many of us are inclined to believe, between the anti-moral acts of madmen like the assassin of the King of the Hellenes and the actions of certain irresponsible men and women in this country whose madness at present stops at arson.

Assassination, no matter what form it takes, is nearly always the result of certain mental processes which, from the standpoint of the average individual, must be considered pathological. How far they are really pathological, science, which has not yet fully accepted the sexual theories of Freud, can scarcely yet determine. The word "assassin" itself is probably of the same derivation as some other words which also concern quasi-pathological states—masochism, burking, and allied terms—for etymologists tell us that it comes from the Arabic and originally meant one who killed cruelly. The legend of the robber captain Hassis, or Assis, is probably the traditional recollection of a particularly severe form of murder mania in a chieftain or headman of a tribe. The fact remains that crimes of the nature of assassination and arson are crimes which depend largely on what Holmes called "intellectual kinks" in the individuals who commit them. Anthropologically, it is interesting to note that these kinks appear to take various forms in different nations. The Teutonic race, on the whole, is averse from either arson or assassination as political weapons. Yet sexual murders and arson as a sexual crime, in the sense that the psycho-analyst uses the term, are increasingly common in Germany, and have of recent years evoked much discussion in scientific circles. With us there can be little question that many of the recent disturbances which have been put down to purely political motives have their real causes in a deeper psychological disturbance than can be accounted for by the annoyance engendered by the non-possession of a vote. Thoughtful analysts of the militant movement have commented on the psychiatry of the women who have identified themselves with acts of violence.

We ourselves believe that some useful purpose might possibly be served by the frank publication of observations made in this direction, although it is true that the public has not been educated to the standard of frankness in regard to these sex-psychiatrical matters that prevails on the Continent. The psychiatrical anamnesis of some of these irresponsible women would at least show the public how utterly unfit these sufferers are to pose as political martyrs, since their acts have all been influenced by circumstances and causes over which they have at present little control. That being the case, it is obvious that the ordinary punishments of the law are powerless to safeguard the public against such irresponsibles. In the case of the assassin the law has at least one resort which, however unscientific and perhaps illogical, does at least prevent the individual from continuing to be a dangerous nuisance by placing him at once beyond the pale of civilisation. No such remedy is possible in the case of the women who commit arson. Their need is curative treatment, not punishment. The proper place for the "hunger-striker," for instance, is the mental hospital, not the prison; and so long as the latter is preferred to the former it is inevitable that the criminal will triumph, simply because the law has hitherto taken no cognisance of the powerful pressure to which the sexual maniac is subject. The very words we are forced to use in dealing with these types of crime show how weak our nomenclature as yet appears in comparison with the better and fuller terminology used abroad. The assassination of the King of the Hellenes and the burning of Lady White's house differ only in this—that the one means the destruction of life and the other that of property; the underlying motive in both cases is the desire to hurt. Cloak that desire as much as they will, the criminals cannot contend that it is otherwise than the primitive impulse and momentary excitement that actuates the maniac, and against which it is the duty of the law to protect the community.

